# Supplementary material for: Optimization of surface roughness for titanium alloy based on multi-strategy fusion snake algorithm
Source: PLoS One. 2025 Jan 16;20(1):e0310365. doi: 10.1371/journal.pone.0310365 (PMC11737802; doi:10.1371/journal.pone.0310365)
Supplement: Attachment — Submitted filename: Response to Reviewers .docx [file pone.0310365.s001.docx]

Dear Dr. Majumder，

Thank you for your letter and for the reviewers’ comments concerning our manuscript entitled “Optimization of Surface Roughness for Titanium Alloy Based on Multi-strategy Fusion Snake Algorithm” (PONE-D-24-20301). Those comments are very valuable and very helpful for revising and improving our paper, as well as the important guiding significance to our researches. We have studied comments carefully and have made correction which we hope meet with approval. In the revision, we have fully addressed the comments made by the reviewers and the editor. The completed changes are as follows:

**Reviewer #Editor:**

**Comment 1**

Please ensure that your manuscript meets PLOS ONE's style requirements, including those for file naming. The PLOS ONE style templates can be found at

<https://journals.plos.org/plosone/s/file?id=wjVg/PLOSOne_formatting_sample_main_body.pdf>

and <https://journals.plos.org/plosone/s/file?id=ba62/PLOSOne_formatting_sample_title_authors_affiliations.pdf>

**Response 1**

The full text format has been revised and checked in plos one style, and I would appreciate your approval.

**Comment 2**

Please note that PLOS ONE has specific guidelines on code sharing for submissions in which author-generated code underpins the findings in the manuscript. In these cases, we expect all author-generated code to be made available without restrictions upon publication of the work.

Please review our guidelines at https://journals.plos.org/plosone/s/materials-and-software-sharing#loc-sharing-code and ensure that your code is shared in a way that follows best practice and facilitates reproducibility and reuse.

**Response 2**

Ok, thanks for the suggestion. We have submitted the code for the article to the public repository：[SO-MS/SO_MS.mlx at main · LiNQ-6688/SO-MS (github.com)](https://github.com/LiNQ-6688/SO-MS/blob/main/SO_MS.mlx).

**Comment 3**

We note that the grant information you provided in the ‘Funding Information’ and ‘Financial Disclosure’ sections do not match.

When you resubmit, please ensure that you provide the correct grant numbers for the awards you received for your study in the ‘Funding Information’ section.

**Response 3**

This issue has been corrected, thank you for your feedback .

**Comment 4**

Thank you for stating the following financial disclosure:

“This study was supported by the National Natural Science Foundation of China (52204169), the project of Liaoning Provincial Science and Technology Department (2023-BS-204) and the project of Liaoning Provincial Department of Education (LJKQZ20222321), and the experimental work in this paper was supported by the Mechanical Processing Centre of Shenyang Casting Research Institute.”

 Please state what role the funders took in the study. If the funders had no role, please state: "The funders had no role in study design, data collection and analysis, decision to publish, or preparation of the manuscript." If this statement is not correct you must amend it as needed.

Please include this amended Role of Funder statement in your cover letter; we will change the online submission form on your behalf.

**Response 4**

This issue has been corrected, thank you for your feedback .

**Comment 5**

Thank you for stating the following in the Acknowledgments Section of your manuscript:

"This study was supported by the National Natural Science Foundation of China (52204169), the project of Liaoning Provincial Science and Technology Department (2023-BS-204) and the project of Liaoning Provincial Department of Education(LJKQZ20222321), and the experimental work in this paper was supported by the Mechanical Processing Centre of Shenyang Casting Research Institute."

Please note that funding information should not appear in the Acknowledgments section or other areas of your manuscript. We will only publish funding information present in the Funding Statement section of the online submission form. Please remove any funding-related text from the manuscript.

**Response 5**

This issue has been corrected, thank you for your feedback .

**Comment 6**

We note that your Data Availability Statement is currently as follows:

"All relevant data are within the manuscript and its Supporting Information files."

Please confirm at this time whether or not your submission contains all raw data required to replicate the results of your study. Authors must share the “minimal data set” for their submission. PLOS defines the minimal data set to consist of the data required to replicate all study findings reported in the article, as well as related metadata and methods (https://journals.plos.org/plosone/s/data-availability#loc-minimal-data-set-definition).

For example, authors should submit the following data:

- The values behind the means, standard deviations and other measures reported;
- The values used to build graphs;
- The points extracted from images for analysis.

Authors do not need to submit their entire data set if only a portion of the data was used in the reported study.

If your submission does not contain these data, please either upload them as Supporting Information files or deposit them to a stable, public repository and provide us with the relevant URLs, DOIs, or accession numbers. For a list of recommended repositories, please see https://journals.plos.org/plosone/s/recommended-repositories.

If there are ethical or legal restrictions on sharing a de-identified data set, please explain them in detail (e.g., data contain potentially sensitive information, data are owned by a third-party organization, etc.) and who has imposed them (e.g., an ethics committee). Please also provide contact information for a data access committee, ethics committee, or other institutional body to which data requests may be sent. If data are owned by a third party, please indicate how others may request data access.

**Response 6**

A change has been made in the submission system to address this issue:“Data and code are available onrequest to the authors.”

**Comment 7**

When completing the data availability statement of the submission form, you indicated that you will make your data available on acceptance. We strongly recommend all authors decide on a data sharing plan before acceptance, as the process can be lengthy and hold up publication timelines. Please note that, though access restrictions are acceptable now, your entire data will need to be made freely accessible if your manuscript is accepted for publication. This policy applies to all data except where public deposition would breach compliance with the protocol approved by your research ethics board. If you are unable to adhere to our open data policy, please kindly revise your statement to explain your reasoning and we will seek the editor's input on an exemption. Please be assured that, once you have provided your new statement, the assessment of your exemption will not hold up the peer review process.

**Response 7**

A change has been made in the submission system to address this issue:“Data and code are available onrequest to the authors.”

**Comment 8**

Please amend the manuscript submission data (via Edit Submission) to include author "Hui Wang".

**Response 8**

Changes have been made in the submission system to address this issue.

**Reviewer #1:**

**Comment 1**

Some key parameters are not mentioned. The rationale on the choice of the particular set of parameters should be explained with more details. Have the authors experimented with other sets of values? What are the sensitivities of these parameters on the results?

**Response 1**

Thank you very much for your comment. When milling titanium alloys at high speed, the optimization of surface roughness is crucial as it directly affects the fatigue strength, corrosion resistance and overall performance of the part. By constructing a high-speed cutting experimental platform and applying Response Surface Methodology (RSM) and Orthogonal Test Method, we were able to systematically investigate the effects of milling parameters on surface integrity.

The experimental results show that spindle speed, width of cut, depth of cut and feed per tooth are the key factors affecting surface roughness. For example, an increase in spindle speed can reduce surface roughness to a certain extent because a higher speed reduces the time the tool is in contact with the material, which in turn reduces plastic deformation and heat accumulation. However, too high a rotational speed may lead to an increase in cutting temperature, affecting tool durability and the surface quality of the workpiece.

Adjustments to the width and depth of cut can optimize the material removal rate, which in turn affects the surface texture and roughness. A proper feed per tooth ensures stability of the cutting process and reduces surface irregularities caused by fluctuations in cutting forces. Through the combined optimization of these parameters, we aim to achieve minimum surface roughness, taking into account productivity and tool life.

In addition, by using a multi-strategy fusion snake algorithm for multi-objective optimization, we are able to improve material removal rates and achieve efficient machining while maintaining surface quality. The selection and optimization of these parameters is not only based on experimental data and statistical analysis, but also takes into account the requirements for machining efficiency and cost-effectiveness in actual production. Through these comprehensive considerations, we are able to provide a theoretical basis and data support for the manufacture of aerospace cast titanium alloy parts, ensuring the reliability of the machining process and the high performance of the parts.

For the parameter sensitivity study, the data show that the depth of cut and feed per tooth have a high sensitivity to surface roughness, while the spindle speed and cutting width, although they also have some influence, are relatively small. By considering these parameters and their interactions in a comprehensive manner, the milling process can be optimized more effectively to achieve surface roughness improvement.

However, the purpose of this paper is how to use the algorithm to select better parameters, and the significance of different parameters for the results has already been reflected in the prediction model to some extent, so it has not been described emphatically.

**Comment 2**

A graphical abstract is needed. It should be compact and to the point.

**Response 2**

We sincerely apologize for overlooking the Graphical summary, with additional graphical summary below.

**Comment 3**

The introduction paragraph should be presented more extensively; many papers were cited, but the outcomes of some of these mentioned papers on optimization were practically neglected. In addition, structure of the paper needs to revise, and all the segments should be well defined. Thus, the Authors are advised to study and incorporate few good papers on optimization as listed below and the Authors need to compare results with the recent metaheuristics if it is applicable. Moreover, the following manuscripts can be added to the list of references, which are in the domain recent algorithms: DOI: 10.32604/cmes.2023.028632; https://doi.org/10.1016/j.mex.2023.102181; https://doi.org/10.1016/j.knosys.2023.110529; DOI: 10.4018/IJAMC.2017070101

**Response 3**

We sincerely apologise for the lack of completeness of the introduction and make a request for additions.z

**Comment 4**

Programming/Codes of the proposed method should be added to reproduce/review the claimed results.

**Response 4**

Ok, thanks for the suggestion. We have submitted the code for the article to the public repository：[SO-MS/SO_MS.mlx at main · LiNQ-6688/SO-MS (github.com)](https://github.com/LiNQ-6688/SO-MS/blob/main/SO_MS.mlx).

**Comment 5**

Authors should argue their choice of the performance evaluation indicators. This paper can be accepted after the implementation of the suggested modification. I suggest revision for the paper.

**Response 5**

Thank you for your advice. Below we will justify the addition of the chosen performance evaluation metrics to the paper.

Surface roughness (Ra): Surface roughness is a key indicator of the surface quality of the part, which directly affects the fatigue life, wear performance and sealing performance of the part. In the high-speed milling process, the optimisation of surface roughness can significantly improve the working performance and reliability of the parts.

**Reviewer #2:**

**Comment 1**

Section 1 (Introduction): It is recommended to add the following reference to the article, which is related to this study in terms of the workpiece material used in the experimental study, machining operation, experimental design and optimization, etc. https://doi.org/10.1016/j.jmapro.2022.06.016

**Response 1**

Thank you very much for your suggestion, we have included more detailed experimental procedures and testing procedures and more reference information in the paper.

**Comment 2**

How were the control factors and levels in Table 1 determined? Please explain.

**Response 2**

We agree with this suggestion and have modified the terminology throughout the text as appropriate.Regarding the selection of control factors, the control factors in Table 1 include spindle speed (*X*_1_), cutting width (*X*_2_), depth of cut (*X*_3_), and feed per tooth (*X*_4_). These factors are key parameters in milling machining, and they directly affect the machining efficiency, surface quality, tool life, and so on. In Tab 1, each control is assigned three levels, which are typically intended for Response Surface Methodology (RSM) or orthogonal experimental designs. These levels represent low, medium, and high settings of the parameter and are used to assess the effect of parameter changes on the response (e.g., surface roughness). A coding of -1, 0, and 1 is used in the table to represent the low, medium, and high levels. This coding is convenient for use in mathematical models, where 0 typically represents the medium level, and -1 and 1 represent settings below and above the medium level, respectively. In response surface analysis, these levels may be determined based on prior literature research, expert opinion, or pre-experimentation to estimate the range of possible effects of the parameters. The goal of the experimental design is to find the optimal combination of parameters to minimise or maximise a given response.The three levels of spindle speed are 500 rpm, 1500 rpm and 2500 rpm. These values are based on the capability of the machine, the recommended speed of the tool and the machining characteristics of the material. The three levels of cutting width are 2 mm, 5 mm and 8 mm, taking into account the size of the workpiece and the required material removal rate. The three levels of depth of cut are 0.1 mm, 0.25 mm and 0.4 mm, depending on the cutting capacity of the tool, the hardness of the workpiece material and the required machining accuracy. The three levels of feed per tooth are 0.01 mm, 0.03 mm and 0.04 mm, which are related to the tool geometry, the machinability of the material and the surface quality required.

**Comment 3**

Are the Ra results in Table 2 the arithmetic average of the surface roughness measurement results? There is a need to explain in more detail how surface roughness measurements are performed in subheading 2.1.

**Response 3**

We agree with this suggestion and have modified the terminology throughout the text as appropriate. we used advanced measurement techniques to evaluate the surface roughness of titanium alloy materials after high-speed milling. Specifically, a PS50 3D surface profiler was used to perform precision surface roughness measurements. This device is based on white light confocal technology, which emits a specific wavelength of light and receives the reflected light back through the probe. By calculating the distance between the probe and the surface, the device is able to accurately acquire the 3D coordinates of the surface. The collected data is analysed in detail using the NANOVIA 3D software to obtain surface roughness measurements from the nanometre to the millimetre scale. The advantages of this measurement method are its high accuracy, fast measurement and good repeatability, providing us with reliable surface roughness data. In addition, in order to further observe the macroscopic morphology of the machined surface, we also used a Japanese Keens VHX-5000 super depth-of-field microscope. This microscope provides a clear image of the surface, helping us to understand the distribution of surface roughness and morphological details more intuitively. By combining these two measurement techniques, we are not only able to obtain quantitative data on surface roughness, but also to conduct in-depth analysis of its micro and macro features, which is of great significance for optimising milling process parameters and improving machining quality.

**Comment 4**

Subheading 2.1: How many times was each experiment repeated? Was a new, unworn cutting edge of the cutting tool used in each test? Please explain these issues.

**Response 4**

Thanks to your suggestion we performed longer milling paths during our experiments to ensure that our results were significant so that only one experiment was needed for each set of parameters.

We thank the reviewers for pointing this out.We really didn't add this detail about the tooling issue In order to minimise the effect of tool wear, new blades were replaced before the start of each set of experiments.

**Comment 5**

Subheading 2.1: What is the chemical composition of Ti64? Write it as text or add it as a table.

**Response 5**

We thank the reviewers for pointing this out. The chemical composition of Ti64 is roughly: Aluminium (Al): 6.20% Vanadium (V): 3.82% Iron (Fe): 0.043% Carbon (C): 0.010% Nitrogen (N): 0.016% Hydrogen (H): 0.0002% Oxygen (O): 0.10%.These chemical compositions together determine the excellent properties of the Ti-6Al-4V alloy.

**Comment 6**

Subheading 2.1: Were the experiments conducted under dry cutting conditions or with coolant? Please explain these issues. (In the photo at the bottom left of Figure 1, it can be seen that the coolant is used, but I am not sure; it cannot be seen clearly.)

**Response 6**

Thank you for your question, we used oil based coolant for this experiment to get better surface roughness.

**Comment 7**

Subheading 2.1, Paragraph 2: "……milling methods are plane milling and inverse milling." "plane milling-->face milling, "inverse milling" --" " up milli"g" o" "down milli"g." Please use the correct technical terms.

**Response 7**

We appreciate your suggested corrections to the academic terminology and will make the corrections as follows I have changed plane milling to face milling and also changed inverse milling to up and down milling.

**Comment 8**

Subheading 2.1. Please specify the technical specifications of the" "APMT1604PDER-"2" insert. Write the cutting tool material. Is the cutting tool tip a cemented carbide tool, coated or uncoated tool? Why was this cutting tool material and geometry chosen for milling the workpiece material? Explain these issues. In this study, much more emphasis was placed on the statistical evaluation of experimental findings. However, to develop the machinability database of the selected workpiece and contribute to the practices in industrial applications, details of machinability experimental studies should also be included in addition to statistical evaluations.

**Response 8**

Thank you to the reviewer for pointing this out. For the technical specifications of APMT1604PDER-M2 blades: Blade shape: Parallelogram top angle 85° Back angle of the main cutting edge: 11 Precision grade: M Chipbreaker groove: Single sided chipbreaker Cutting edge length: 15.875mm Blade thickness: 4.76mm Main offset angle of the main cutting edge: 90° Back angle of the trimming edge: 15°, and for the cutting tool material : The cutting tool material is carbide with external coating. For tip type: The tips are coated carbide tools. Coatings are often used to improve tool durability and performance, reduce friction and increase wear resistance. Then for the reasons of choosing cutting tool material and geometry: one of them is Cemented Carbide: Cemented Carbide is widely used in cutting tools due to its high hardness, wear resistance and high thermal stability. It can effectively withstand the heat and mechanical loads generated during high-speed milling. Secondly Coatings: Coatings can further enhance the performance of carbide tools by reducing the wear by reducing the coefficient of friction between the tool and the work material and also provide better chemical stability and oxidation resistance. Thirdly Geometry: The insert geometry (e.g. top angle, back angle, etc.) is selected to suit the cutting characteristics of the titanium alloy material. For example, a proper back angle reduces the contact area between the tool and the chip, thus reducing heat generation and propagation, while the main deflection angle affects the distribution of cutting forces. Statistical evaluation and experimental study of machinability: In this study, we conducted a multi-objective optimisation study of high-speed milling parameters of aerospace cast titanium alloys by means of experimental tests, statistical analysis and artificial intelligence algorithms. Statistical evaluation is important because it provides a quantitative understanding of the effect of milling parameters on surface integrity. In order to develop a machinability database for titanium alloys and to facilitate industrial applications, a detailed experimental study of machinability should be included in addition to the statistical evaluation. This includes tool material selection, optimisation of tool geometry, determination of cutting parameters (e.g. cutting speed, feed rate and depth of cut) and their influence on machining efficiency, surface quality and tool life. Statistical evaluation of experimental results: the statistical evaluation of experimental results is carried out by methods such as Response Surface Methodology (RSM) and Analysis of Variance (ANOVA). These methods help the researcher to understand the effect of different milling parameters on surface roughness and material removal rate and to determine the optimum combination of parameters.

**Comment 9**

Subheading 2.1: As seen in Table 1, in this experimental study, milling parameters (for example, cutting speed, feed amount, cutting depth, cutting width) were selected as input parameters. However, titanium alloy materials are among the materials that are difficult to process (for example, mill). Besides milling parameters, control factors such as cutting tool material, geometry and coating layer, and cooling environment (e.g., dry and MQL) also affect the output parameters (machinability parameters). Why were these parameters not selected as control factors apart from the selected milling parameters? Authors should also explain these issues.

**Response 9**

Thanks to the reviewers for pointing this out, the main objective of the research in this paper may have focused on improving surface integrity by optimizing milling parameters. Therefore, the research design may have intentionally kept other variables under control in order to more clearly observe and quantify the effects of milling parameters. Adding more controls significantly increases the complexity of the experimental design, resulting in the need for more experiments to fully cover all possible parameter combinations, which may not be feasible in terms of time and resources. The use of different types of tool materials, geometries and coatings, as well as changes in the cooling environment, may increase the cost of the experiment, especially if expensive tools or special cooling techniques are used. In order to ensure reproducible and comparable experimental results, we chose at that time to conduct our experiments under standardized conditions, e.g. using a single type of tool and a fixed cooling method. Based on the existing literature and knowledge, which has shown that certain tool materials and geometries are better for milling titanium alloys, these were chosen as fixed parameters to establish a reliable benchmark. A stepwise optimization strategy was adopted whereby the main milling parameters were optimized first and then the effects of other control factors were considered in subsequent studies. Experimental equipment and capabilities may limit the number of variables that can be controlled simultaneously. For example, some equipment may only be suitable for specific tool types or cooling methods. Having too many control factors may complicate the interpretation of experimental results and make it difficult to clearly correlate to specific input parameters. The study may be designed to simulate real-world conditions in industrial production, where certain factors (such as tool materials and coatings) may be fixed due to cost, availability or other considerations.

**Comment 10**

Subheading 2.1: It has been stated that surface roughness were measured with a 3D microsystem. In 2D measurements using the profile meter method, Ra values are generally measured and evaluated, and the unit is a micrometer. In 3D measurements, Sa, not Ra, is measured. What is the unit of Sa measurements? Please review and analyze these evaluations.

**Response 10**

Thanks to the reviewer for pointing this out, the 3D surface topography measurements mentioned in this paper were made using a PS50 3D surface topographer, which uses a white light confocal technique for the measurements. In 3D measurements, a commonly used parameter is Sa (Arithmetic Mean Height), which is similar to Ra (Arithmetic Mean Roughness), but Sa is a parameter for 3D surface measurements, whereas Ra is usually used for 2D measurements. Sa is calculated by summing up the absolute value of the deviation of the heights of all the measured points from their mean heights and dividing by the absolute value of the deviation of the heights of all the measured points from their mean heights. Sa is calculated by summing the absolute value of the deviation of the heights of all the measured points from their average height and dividing by the total number of measured points. Sa provides an overall measure of the surface roughness, including both height and magnitude information.

Units of measurement for Sa: Like Ra, Sa is usually measured in microns (μm) because they are both parameters used to measure the micro-geometric characteristics of surface roughness. A clear distinction is made between Ra and Sa because measurements are made in three dimensions. In practice, three-dimensional measurements provide more comprehensive information about surface topography, including peak and valley heights, valley depths, and peak densities, which are all important parameters for assessing the surface integrity of a part.

**Comment 11**

Subheading 2.1: Apart from surface roughness, cutting forces, tool life, tool wear, chip type, etc. Why were the output parameters/variables not evaluated in this study? Is only evaluating the surface roughness sufficient to evaluate the machinability of titanium alloy? It is recommended that authors examine and evaluate these issues.

**Response 11**

We thank the reviewers for pointing this out and indeed there are other factors that may affect the machinability of titanium alloys, it is due to the depth of the subject matter and the wide range of disciplines involved that the assessment of surface roughness alone is not sufficient for a comprehensive evaluation of the machinability of titanium alloys due to the limitations of the experimental conditions and knowledge base. Although surface roughness is one of the most important indicators of machining quality, which affects the appearance, fatigue life and corrosion resistance of the part, machinability is a comprehensive concept involving several aspects may involve residual stresses. We will address residual stresses and machining distortion in a subsequent study.

**Comment 12**

There is a problem in the organization of the subheadings (chapters) of the article. Table 2 in Subheading 2.2 and Tables 3-5 in Subheading 2.3 should be presented under the newly created Section 3. (Experiment and experimental analysis or Result and Discussions) heading. Section 3 and Section 4 should be given as subheadings of Section 2. If the sections of the article, namely subheadings, are arranged, the article will be more organized, followable, and motivating. The information provided in these sections is very useful and necessary; only the order and organization of the subheadings are problematic, and their revision is strongly recommended.

**Response 12**

We apologize for the confision generated by the previous version of the manuscript and sincerely hope that our logic is now easier to follow with this new version.

**Comment 13**

Subheading 2.2: Has the procedure in RSM been applied to determine the levels of the milling parameters given in Table 1? Prove that logarithmic calculation in RSM is used to determine the levels of milling parameters. As the article's authors, please review the calculations to determine the levels of the input parameters (independent variables) in RSM.

**Response 13**

Thanks to the reviewers for pointing this out, we used RSM to optimize the parameters for high-speed milling of titanium alloys, including spindle speed, width of cut, depth of cut and feed per tooth. These parameters are the input independent variables, while surface roughness and material removal rate are the response variables.

**Comment 14**

Subheading 3, Subheading 4: Please refer to the explanations and information about the techniques, methods, algorithms, and equations given under these subheadings and indicate the reference numbers in the relevant texts in the article. Because many equations and methods given under these headings were not developed by the article's authors.

**Response 14**

Changes have been made to address this issue, thank you for your suggestion.

**Comment 15**

Explain the meanings of parameters, variables, constants, subscripts, and superscripts used in the equations in the article immediately after the relevant equations. Preparation of a Nomenclature for this article is highly recommended.

**Response 15**

Thank you very much for pointing this out, we have included a glossary of terms for you below!

| **Nomenclature** |  |
| --- | --- |
| *X*_1、_*X*_2、_*X*_3、_*X*_4_ | Milling parameters: spindle speed, width of cut, depth of cut, feed per tooth |
| *Ra* | surface roughness |
| $\beta_{0},\beta_{s},\beta_{ss},\beta_{sd}$ | Regression coefficients: constant term, primary term coefficient, squared term coefficient, interaction term coefficient |
| *Q* | Total amount of food, snake algorithm parameters |
| *Temp* | Temperature parameters, snake algorithm control parameters |
| *gbest* | global optimum solution |
| *i,j* | Different factors or levels in a multifactor test |
| *m,f* | Individual male and female snakes |
| *t* | Number of iterations or time steps |
| R^2^ | coefficient of determination |
| Adjusted R^2^ | Adjusted coefficient of determination |
| Predicted R^2^ | Projected coefficient of determination |

**Comment 16**

The visuality and resolution of Figure 3 should be increased. Authors can re-draw the figure in Figure 3 themselves.

**Response 16**

Thank you for your comments on the image, we will work on improving the visibility and resolution of Figure 3

**Comment 17**

To better understand the sequence, steps, and interrelationships of the experimental studies and statistical evaluations performed in this article, prepare a detailed flowchart and attach it to Subheading 2. It is thought that a flowchart prepared to summarize the work done in the article and reflect the steps followed will contribute greatly to the article.

**Response 17**

Thank you for pointing out the problem, and in order to better understand the sequence, steps and interrelationships of the experimental studies and statistical evaluations carried out in this paper, we have attached a detailed flowchart.

**Comment 18**

Section 2.3: ANOVA is given in Table 4 for the predictive models developed in this study. However, in addition to the ANOVA given in Tab 4, the article should also add an ANOVA reflecting the main effects of milling parameters on Ra and the effects of interactions. According to this ANOVA table to be added, the statistically effective milling parameters and their % contribution on Ra should be stated. At the same time, the effects of milling parameters on Ra should be explained in terms of machinability research by comparing similar research results in the literature. In other words, explaining the results obtained from evaluating the experimental findings with statistical methods does not provide much depth or extra contribution to the article.

**Response 18**

We add the interpretation of the ANOVA results: the linear mode has an F-value of 35.62 and a P-value of 0.0001, which is much less than 0.05, indicating that at least one of the milling parameters in the model has a significant main effect on Ra. Also spindle speed (X_1)_, width of cut (X_2_), depth of cut (X_3_) and feed per tooth (X_4_) as components of the linear model have a direct effect on Ra. Since the p-values are not given, we cannot determine the specific significance of each parameter, but the significance of the overall linear model suggests that these parameters, at least in general, have a significant effect on Ra. From the interaction effect analysis, the F-value regarding the quadratic model was 16.63 with a p-value of 0.0001, indicating that the interaction effect between the milling parameters also had a significant effect on Ra. This may include interactions between parameters such as X_1_X_2,_ X_1_X_3_, X_1_X_4,_ X_2_X_3_, X_2_X_4_ and X_3_X_4_.The contribution can be assessed by the R² value of the model, which indicates the amount of variance explained by the model. Comparing the results of similar studies in the literature can help us understand the milling characteristics of the titanium alloy Ti-6Al-4V. Other studies may have reported Ra values for different materials or different cutting conditions, and these data can be used to compare and validate the findings of this study. The effect of milling parameters on Ra, from the machinability point of view, the milling parameters listed as cutting speed, feed rate and depth of cut directly affect the material removal rate and heat generation, which in turn affects the surface roughness. Higher cutting speeds may lead to higher cutting temperatures, which in turn affects the plastic deformation of the material and Ra. It is also recognised that statistical methods, although capable of revealing the effect of milling parameters on Ra, may not be able to fully account for all the observed phenomena. Factors such as the microstructure of the material, tool wear, and the cooling effect of the cutting fluid may have an important effect on Ra, but these factors may not be adequately represented in the statistical models. Regarding additional contributions in order to provide deeper insights, studies could combine experimental results and theoretical analyses to explore how milling parameters can change Ra by affecting the material removal mechanism.

All authors have read and approved the re-submission of the manuscript! If you have any questions, please let me know!

Thank you for your consideration of our paper and we are looking forward to hearing from you!

Sincerely yours,

Zuen Shang
