# Supplementary material for: Optimization of surface roughness for titanium alloy based on multi-strategy fusion snake algorithm
Source: PLoS One. 2025 Jan 16;20(1):e0310365. doi: 10.1371/journal.pone.0310365 (PMC11737802; doi:10.1371/journal.pone.0310365)
Supplement: Attachment — Submitted filename: Response to Reviewers.docx [file pone.0310365.s002.docx]

Dear Dr. Majumder，

Thank you for your letter and for the reviewers’ comments concerning our manuscript entitled “Optimization of Surface Roughness for Titanium Alloy Based on Multi-strategy Fusion Snake Algorithm” (PONE-D-24-20301). Those comments are very valuable and very helpful for revising and improving our paper, as well as the important guiding significance to our researches. We have studied comments carefully and have made correction which we hope meet with approval. In the revision, we have fully addressed the comments made by the reviewers and the editor. The completed changes are as follows:

**Comment 1**

The authors addressed all the reviewers' comments. There are only some misprints that should be fixed. They are indicated in the following:

The word "we" at the beginning of a paragraph after figure 1, in the sentence "we used advanced measurement techniques…" should be capitalized since it is at the beginning of the phrase.

Please consider the same suggestion for the beginning of the phrase "the linear mode has an F-value of …" of the paragraph after table 5. In addition, there is a misprint in this paragraph related to the word "overall" at the fifth raw.

Please capitalize the first letter of the title of subsection 2.4 Regression model.

**Response 1**

Thank you very much for your comment. Spelling errors have been corrected and labelled in accordance with the reviewer's comments.

All authors have read and approved the re-submission of the manuscript! If you have any questions, please let me know!

Thank you for your consideration of our paper and we are looking forward to hearing from you!

Sincerely yours,

ZuEn Shang
